# Probing Surface Morphology using X-ray Grating Interferometry

**DOI:** 10.1038/s41598-019-50486-5

**Published:** 2019-10-01

**Authors:** Wataru Yashiro, Susumu Ikeda, Yasuo Wada, Kentaro Totsu, Yoshio Suzuki, Akihisa Takeuchi

**Affiliations:** 1 Institute of Multidisciplinary Research for Advanced Materials (IMRAM), Tohoku University, 2-1-1 Katahira, Aoba-ku, Sendai, Miyagi 980-8577 Japan; 2 WPI-Advanced Institute for Materials Research (WPI-AIMR), Tohoku University, 2-1-1 Katahira, Aoba-ku, Sendai, Miyagi 980-8577 Japan; 3 Faculty of Science and Technology, Keio University, 3-14-1 Hiyoshi, Kohoku-ku, Yokohama, Kanagawa 223-8522 Japan; 4 Micro System Integration Center (μSIC), Tohoku University, 519-1176 Aramaki-Aza-Aoba, Aoba-ku, Sendai, Miyagi 980-0845 Japan; 5 Japan Synchrotron Radiation Research Institute (JASRI), 1-1-1 Kouto, Sayo-cho, Sayo-gun, Hyogo, 679-5198 Japan

**Keywords:** Imaging techniques, Imaging and sensing

## Abstract

X-ray reflectometry (XRR), a surface-sensitive technique widely used for characterizing surfaces, buried interfaces, thin films, and multilayers, enables determination of the electron density distribution perpendicular to a well-defined surface specularly reflecting X-rays. However, the electron density distribution parallel to the surface cannot be determined from an X-ray reflectivity curve. The electron density correlation in the lateral direction is usually probed by measuring the grazing-incidence small-angle X-ray scattering (GISAXS). GISAXS measurement, however, typically requires using a collimated X-ray point beam to distinguish the GISAXS from the specularly reflected X-rays, and so the sample must be scanned in the lateral direction with the point beam to investigate variations in the surface and interface morphology for a region larger than the size of the beam. In this paper, we report a new approach based on X-ray grating interferometry: an X-ray sheet beam is used instead of an X-ray point beam. A method using this approach can simultaneously provide one-dimensional real-space images of X-ray reflectivity, surface curvature, and ‘dark-field’ contrast with a field-of-view of more than a few millimetres. As a demonstration, a sample having a 400 nm line and space SiO_2_ pattern with a depth of 10 nm on its surface was used, and the dark-field contrast due to the unresolved line and space structure, creating GISAXS in the lateral direction, was successfully observed. Quantitative analysis of these contrasts provided the real-space distribution of the structural parameters for a simple model of the grating structure. Our study paves the way to a new approach to structure analysis, providing a quantitative way to investigate real-space variations in surface and interface morphology through wavefront analysis.

## Introduction

X-ray reflectometry (XRR) is a standard tool for characterizing surfaces, buried interfaces, thin films, and multilayers in various fields of materials science^1–3^. It is used to measure an X-ray reflectivity curve, *i*.*e*. glancing-angle dependence of the intensity of specularly reflected X-rays with a scattering vector perpendicular to the sample surface, and thereby determine the electron density distribution in the depth direction. Since the scattering vector has no component parallel to the surface, the electron density distribution in the lateral direction cannot be determined from the X-ray reflectivity curve. To estimate the correlation of the electron density near the surface in the lateral direction, small-angle X-ray scattering (SAXS) having a finite in-plane scattering vector component is measured in the grazing-incidence geometry, which makes the SAXS surface sensitive.

This grazing-incidence small-angle X-ray scattering (GISAXS) technique^2–6^ was introduced by Levine *et al*.^4^. The intensity distributions of GISAXS arising around the specularly reflected X-rays are recorded with an image detector and mapped in reciprocal space. However, both the size and angular spread of the X-ray beam normally have to be small enough to distinguish the GISAXS from the specularly reflected beam. Therefore, the sample must be scanned in the lateral direction with a collimated X-ray point beam, the size of which is typically less than submillimeter, to investigate variations in the surface and interface morphology for a region larger than the beam size.

In this paper, we report a novel approach for investigating surface and interface structural parameters through wavefront analysis based on X-ray grating interferometry^7–12^. With this approach, an X-ray sheet beam is used instead of an X-ray point beam, and three independent one-dimensional images can be provided: an X-ray reflectivity image, a surface curvature image, and dark-field-contrast image. The last one provides information on structural parameters that are inferred from GISAXS measurements.

X-ray grating interferometry, an X-ray phase-contrast imaging technique^13–15^, has attracted increasing interest over the last decade because it not only enables X-ray imaging with higher sensitivity than does conventional X-ray absorption-contrast imaging, but also works with a compact laboratory X-ray source. Another advantage of this interferometry is its multi-modality: it provides three independent images^11^ (absorption, differential-phase, and dark-field (more generally, visibility-contrast^16^) images) from a series of experimentally obtained images. The dark-field contrast can be quantitatively related to the angular distribution of SAXS from unresolvable microstructures, the sizes of which are typically of the order of *μ*m^12,17^.

**Figure 1:**
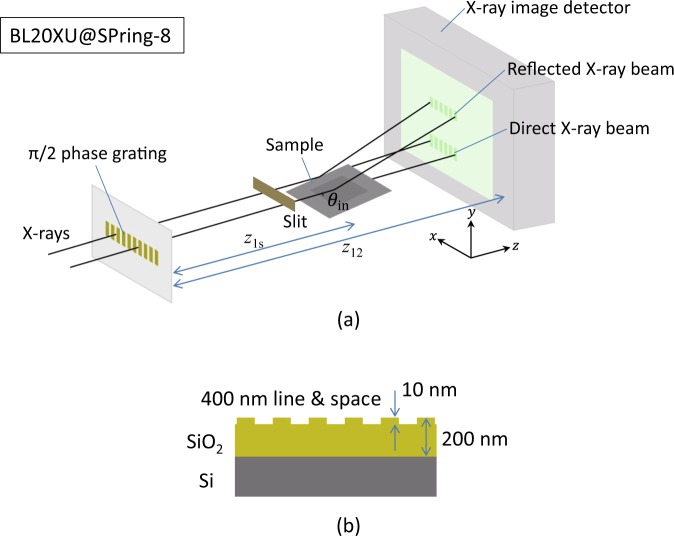
(**a**) Experimental setup for grazing-incidence small-angle X-ray scattering (GISAXS) imaging using an X-ray grating interferometer (*z*_1s_: distance of sample from phase grating (≡*R*_s_ − *R*_1_), *z*_12_: distance between phase grating and X-ray image detector, S1: slit). (**b**) Sample used; surface had 400 nm line and space SiO_2_ pattern.

We applied this multi-modal X-ray imaging technique to structure analysis in the grazing-incidence geometry. Our experimental setup is shown in Fig. 1(a). We constructed an X-ray grating interferometer consisting of a phase grating and an X-ray image detector. The phase grating, the lines of which were aligned in the vertical direction (*y*-direction), was illuminated by an X-ray sheet beam with a sufficiently high spatial coherence in the horizontal direction (*x*-direction) so that a one-dimensional periodic pattern (a self-image^18^) due to the Talbot effect was produced on the detector. A sample 12 mm × 12 mm Si wafer covered with a 200 nm SiO_2_ layer having a 400 nm line and space pattern with a depth of 10 nm in an area of 5 mm × 5 mm on it^19^ (see Fig. 1(a,b)) was positioned between the phase grating and detector. The sample surface was aligned so that the lines were parallel to the optical axis (*z*-axis) when glancing angle *θ*_in_ = 0, and the sheet beam specularly reflected by the sample surface was captured by the detector.

Similar to conventional X-ray grating interferometry, a fringe scanning method^20^ was applied to self-images on the specularly reflected sheet beam^21^. By a fringe scanning method, we can obtain the 0th and 1st order Fourier components of the intensity of the self-image. The 0th order Fourier component corresponds to the average intensity of the self-image, while the phase of the 1st order Fourier component corresponds to the phase of the self-image, which is sensitive to the propagation direction of X-rays. The visibility of the self-image, which is proportional to the ratio of the modulus of the 1st order Fourier component to that of the 0th order Fourier component, is reduced by fluctuation of the phase of X-ray wave due to unresolvable microstructures^12^.

For later convenience, we express by equations the images we will obtain for surface and interface structure analysis. Since a self-image on a specularly reflected sheet beam has one-dimensional periodicity in the *x*-direction, the intensity of the self-image, *I*_self_, can be expanded into a Fourier series:

1 $${I}_{{\rm{self}}}={\Sigma }_{n}{a}_{n}\,\exp [2\pi i\frac{nx}{d}],$$

where $n=0,\pm \,1,\pm \,2,\ldots ,d$ is the pitch of the self-image. Fourier coefficient *a*_*n*_ can be obtained at each pixel using fringe scanning. From the 0th and 1st order Fourier coefficients of the direct beam without the sample, ${a}_{0}^{{\rm{direct}}}$ and ${a}_{1}^{{\rm{direct}}}$, and those of the reflected beam with the sample, ${a}_{0}^{{\rm{reflected}}}$ and ${a}_{1}^{{\rm{reflected}}}$, we can obtain three images^11^:

2 $$ {\mathcal I} (x,{y}_{{\rm{c}}}+\Delta y)=\frac{{a}_{0}^{{\rm{reflected}}}(x,{y}_{{\rm{c}}}+\Delta y)}{{a}_{0}^{{\rm{direct}}}(x,{y}_{0{\rm{c}}}-\Delta y)},$$

3 $${\mathscr{P}}(x,{y}_{{\rm{c}}}+\Delta y)={\rm{\arg }}[\frac{{a}_{1}^{{\rm{reflected}}}(x,{y}_{{\rm{c}}}+\Delta y)}{{a}_{1}^{{\rm{direct}}}(x,{y}_{0{\rm{c}}}-\Delta y)}],$$

4 $${\mathscr{V}}(x,{y}_{{\rm{c}}}+\Delta y)=\frac{{v}^{{\rm{reflected}}}(x,{y}_{{\rm{c}}}+\Delta y)}{{v}^{{\rm{direct}}}(x,{y}_{0{\rm{c}}}-\Delta y)},$$

where *y*_0*c*_ and *y*_*c*_ are the y-coordinates at the centres of the direct and reflected beams (Δ*y* expresses the deviation of the y-coordinate from *y*_*c*_) and *v*^direct^ and *v*^reflected^ are the visibilities of their self-images, defined by $2|{a}_{1}^{{\rm{reflected}}}|/{a}_{0}^{{\rm{reflected}}}$ and $2|{a}_{1}^{{\rm{direct}}}|/{a}_{0}^{{\rm{direct}}}$.

Since the 0th order Fourier coefficient corresponds to the average intensity of the self-image, image ${\mathcal I}$ in Eq. (2) can be interpreted as X-ray reflectivity. Image ${\mathscr{P}}$ is proportional to the wavefront gradient of the reflected X-rays, from which the local curvature of the surface reflecting X-rays can be obtained^22–24^. Image ${\mathscr{V}}$ is referred to as normalised visibility, and can be approximately expressed by a normalised auto-correlation function at two points separated by *p*_s_*d*_1_^12^. Here, *p*_s_ is an effective Talbot order^17,25^ geometrically defined by

5 $${p}_{{\rm{s}}}\equiv \{\begin{array}{ll}{p}_{12}\cdot \frac{{R}_{{\rm{s}}}}{{R}_{1}} & ({R}_{{\rm{s}}}\le {R}_{1})\\ {p}_{12}\cdot \frac{{R}_{2}-{R}_{{\rm{s}}}}{{R}_{2}-{R}_{1}} & ({R}_{{\rm{s}}}\ge {R}_{1})\end{array},$$

where *p*_12_ is the Talbot order for the self-image of the phase grating generated on the detector, and *d*_1_ is the pitch of the phase grating (*R*_s_, *R*_1_, and *R*_2_ are the distances of the sample, the phase grating, and the detector from the X-ray source). Since *p*_s_ depends on *R*_s_, we can change *p*_s_ by changing the position of the sample. The Fourier transform of ${\mathscr{V}}$ with respect to *p*_s_*d*_1_ corresponds to the angular distribution of SAXS intensity^12^ when the origin of the reduction in ${\mathscr{V}}$ is SAXS.

**Figure 2:**
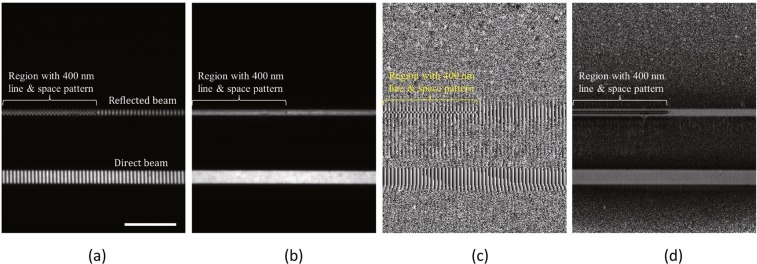
(**a**) Experimentally obtained self-image on specularly reflected sheet beam for ${p}_{{\rm{s}}}{d}_{1}=400\,{\rm{nm}}$ at a glancing angle of 0.195° (gray scale: 0-40000 ADU). (**b**–**d**) Three images obtained using 5-step equal sampling fringe scanning method ((**b**) average intensity of self-images (gray scale: 0-27000 ADU), (**c**) phase of self-images (gray scale: −*π*-*π*), and (**d**) visibility of self-images (gray scale: 0–1.2)). Scale bar: 500 *μ*m.

Figure 2(a) shows an example self-image on a specularly reflected sheet beam obtained with an exposure time of 300 ms for ${p}_{{\rm{s}}}{d}_{1}=400\,{\rm{nm}}$ at a glancing angle of 0.195°, which is close to the critical angle of the total external reflection of the surface. The left-hand side in the figure corresponds to the region with the line and space pattern and the right-hand side corresponds to that without the pattern. A *π*-phase shift of the self-image is evident in the region with the pattern. This shift is qualitatively explained by the ±1st-order diffraction waves arising from the line and space pattern working as a phase grating with a phase shift close to *π*. Figure 2(b–d) show three images obtained from 5 self-images by the 5-step equal sampling fringe scanning algorithm^20^ with a total exposure time of 300 ms × 5 ((b) the average intensity of the self-images, (c) the phase of the self-images, and (d) the visibility of the self-images, corresponding to ${a}_{0}^{{\rm{reflected}}}$, ${\rm{\arg }}({a}_{1}^{{\rm{reflected}}})$, and $2|{a}_{1}^{{\rm{reflected}}}|/|{a}_{0}^{{\rm{reflected}}}|$, respectively).

**Figure 3:**
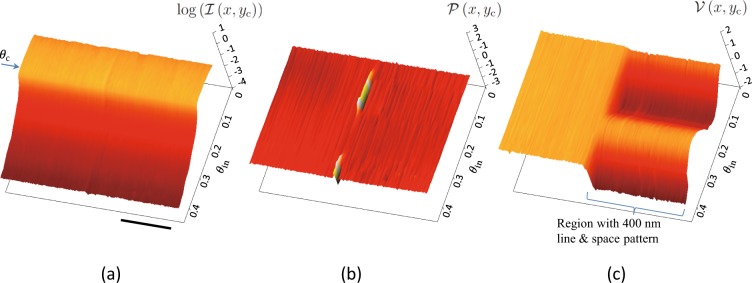
Three-dimensional surface plots of glancing-angle dependences of (**a**) $\log ( {\mathcal I} (x,{y}_{{\rm{c}}}))$, (**b**) ${\mathscr{P}}(x,{y}_{{\rm{c}}})$, and (**c**) ${\mathscr{V}}(x,{y}_{{\rm{c}}})$. In (**a**), *θ*_c_ indicates critical angle for total external reflection. Scale bar: 500 *μ*m.

We measured the glancing-angle dependences of the three images for ${p}_{{\rm{s}}}{d}_{1}=400\,{\rm{nm}}$ and found that the visibility image sensitively changes with an increase in the glancing angle (see Supplementary Movie 1). Figure 3 shows three-dimensional surface plots of the glancing-angle dependences of $\log ( {\mathcal I} (x,{y}_{{\rm{c}}}))$, ${\mathscr{P}}(x,{y}_{{\rm{c}}})$, and ${\mathscr{V}}(x,{y}_{{\rm{c}}})$ with a total exposure time of 31.5 s at each glancing angle. We defined ${\mathscr{V}}$ as taking a negative value and unwrapped ${\mathscr{P}}$ when $\pi /2\le {\mathscr{P}} < \pi$ or $-\,\pi \le {\mathscr{P}} < -\,\pi /2$ to avoid *π*-wrapping in the ${\mathscr{P}}$ plot. The result of this *π*-unwrapping showed almost flat ${\mathscr{P}}$ in Fig. 3(b) except for close to the pattern boundary, which is consistent with the result of surface curvature measurement using a three-dimensional optical profiler (Supplementary Note Section 2) showing a radius of curvature of 2 × 10^2^ m. In Fig. 3(a), no clear difference is seen between the regions with and without the line and space pattern (right- and left-hand sides, respectively), but clear contrast is seen in Fig. 3(c): no clear interference fringe was observed in the X-ray reflectivity while clear fringes, which were expected to reflect the surface structure in the lateral direction, were observed in the normalised visibility.

**Figure 4:**
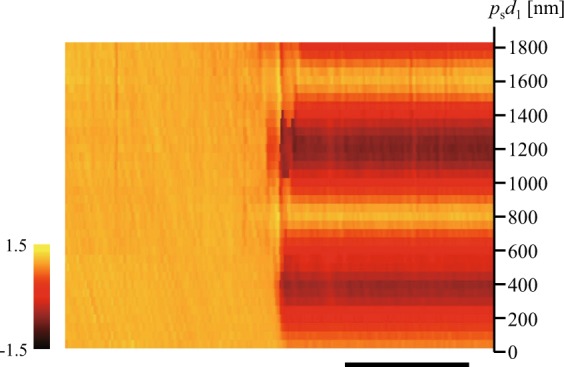
*p*_s_*d*_1_ dependence of ${\mathscr{V}}(x,{y}_{{\rm{c}}})$ at a glancing angle of 0.180°. Scale bar: 500 *μ*m.

We also measured the *p*_s_*d*_1_ dependence of the surface plot of ${\mathscr{V}}(x,{y}_{{\rm{c}}})$ (Supplementary Movie 2). Figure 4 shows an example of the *p*_s_*d*_1_ dependence of ${\mathscr{V}}(x,{y}_{{\rm{c}}})$ at a glancing angle of 0.180°, where the contrast of ${\mathscr{V}}$ was most clearly observed. In the region with the line and space pattern, 800 nm-periodic contrast is evident. This period corresponds to the pitch of the 400 nm line and space pattern in the lateral direction.

**Figure 5:**
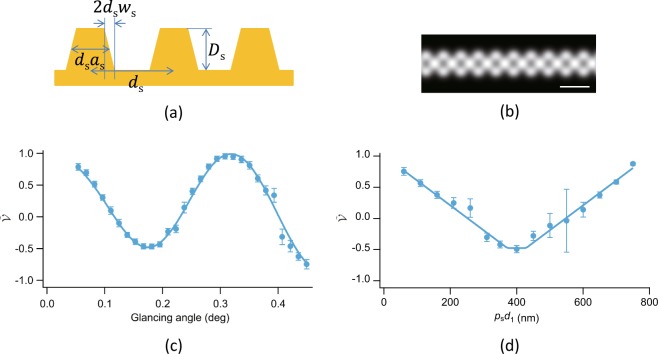
(**a**) Structural model of sample. (**b**) Simulated self-image for ${p}_{{\rm{s}}}{d}_{1}=400\,{\rm{nm}}$ at glancing angle of 0.195° (scale bar: 50 *μ*m). (**c**) Example of least-squares fitting to glancing-angle dependence of $\tilde{{\mathscr{V}}}(x,{y}_{{\rm{c}}})$ for ${p}_{{\rm{s}}}{d}_{1}=400\,{\rm{nm}}$ (filled circles: experimental data, solid line: best-fit curve). (**d**) Example of least-squares fitting for *p*_s_*d*_1_ dependence of $\tilde{{\mathscr{V}}}(x,{y}_{{\rm{c}}})$ for a glancing angle of 0.180° (filled circles: experimental data, solid line: best-fit curve). In (**c**,**d**), error bars were determined using priviously published method^43^ and curves were calculated on basis of rigorous theoretical description^16^.

To explain the experimentally obtained results, we constructed a simple structural model for the sample shown in Fig. 5(a), where *d*_s_ and *D*_s_ are the pitch and depth of the line and space pattern, and ${d}_{{\rm{s}}}{a}_{{\rm{s}}}$ and $2{d}_{{\rm{s}}}{w}_{{\rm{s}}}$ correspond to the average widths of the line and slope ($0\le {a}_{{\rm{s}}}\le 1$ and $0\le 2{w}_{{\rm{s}}}\le \,\min ({a}_{{\rm{s}}},1-{a}_{{\rm{s}}})$). We simulated the experimentally obtained self-image for the reflected X-ray beam in the projection approximation^26^, taking into account the effect of a finite full width at half maximum (FWHM) *W*_D_ of the point spread function (PSF) of the detector. Figure 5(b) shows an example simulated self-image for ${p}_{{\rm{s}}}{d}_{1}=400\,{\rm{nm}}$ at a glancing angle of 0.195° (${d}_{{\rm{s}}}=800\,{\rm{nm}}$, ${D}_{{\rm{s}}}=12\,{\rm{nm}}$, ${a}_{{\rm{s}}}=0$, ${w}_{{\rm{s}}}=0$, *W*_D_ = 14.6 *μ*m), which well reproduces the result shown in Fig. 2(a). Here, the spatial coherence length^25^ on the phase grating was empirically determined to be 20.7 *μ*m. Note that the effect of Fresnel diffraction caused by the sample on the simulated image was negligible; this is consistent with a previous report^16^.

To refine the structural parameters, we performed least-squares fittings to the experimentally obtained results. To eliminate the effect of parasitic X-ray scattering, we defined renormalised visibility $\tilde{{\mathscr{V}}}$ as normalised visibility that is further normalised by the average normalised visibility in the region without the line and space pattern, and performed least-squares fitting to $\tilde{{\mathscr{V}}}(x,{y}_{{\rm{c}}})$ rather than to ${\mathscr{V}}(x,{y}_{{\rm{c}}})$. The normalised visibility was calculated on the basis of a rigorous theoretical description^16^ except that projection approximation was used to reduce computing time. Because the glancing-angle dependence of the normalised visibility is sensitive to *D*_s_, *a*_s_, *w*_s_, and *W*_D_, and the ${p}_{{\rm{s}}}{d}_{1}$ dependence is sensitive to *d*_s_, *a*_s_, *w*_s_, and *W*_D_ (Supplementary Note Section 1), *D*_s_ and *d*_s_ were mainly determined by the glancing-angle and ${p}_{{\rm{s}}}{d}_{1}$ dependences, respectively, while the other parameters were determined by both dependences.

Figure 5(c,d) present the results of least-squares fitting to the glancing-angle dependence of $\tilde{{\mathscr{V}}}(x,{y}_{{\rm{c}}})$ for ${p}_{{\rm{s}}}{d}_{1}=400\,{\rm{nm}}$ and the ${p}_{{\rm{s}}}{d}_{1}$ dependence of $\tilde{{\mathscr{V}}}(x,{y}_{{\rm{c}}})$ at a glancing angle of 0.180° at 500 *μ*m from the edge of the line and space pattern, where the filled circles represent the experimental data and the solid line is the best-fit curve (${d}_{{\rm{s}}}=798\pm 10\,{\rm{nm}}$, ${D}_{{\rm{s}}}=12.4\pm 0.1\,{\rm{nm}}$, ${a}_{{\rm{s}}}=0.462\pm 0.006$, ${w}_{{\rm{s}}}=0.000+0.004$, and *W*_D_ = 13.6 ± 0.4 *μ*m). The reduced ${\chi }^{2}$ for the best-fit curve was 0.87. Note that the fringe period of the glancing-angle dependence of $\tilde{{\mathscr{V}}}(x,{y}_{{\rm{c}}})$ in Fig. 5(c) mainly determines *D*_s_. The fringes appear to be similar to the Kiessig fringes in X-ray reflectivity curves, but have a different origin: $\tilde{{\mathscr{V}}}$ at a glancing angle is determined by the autocorrelation in the lateral direction for a given momentum transfer component normal to the sample surface. In fact, no clear Kiessig fringes were observed in X-ray reflectivity data (see Fig. 3(a) and Supplementary Note Section 3) in the region with the line and space pattern; the observed X-ray reflectivity was consistent with that calculated for a flat surface of SiO_2_ (2.3 g/cm^3^).

**Figure 6:**
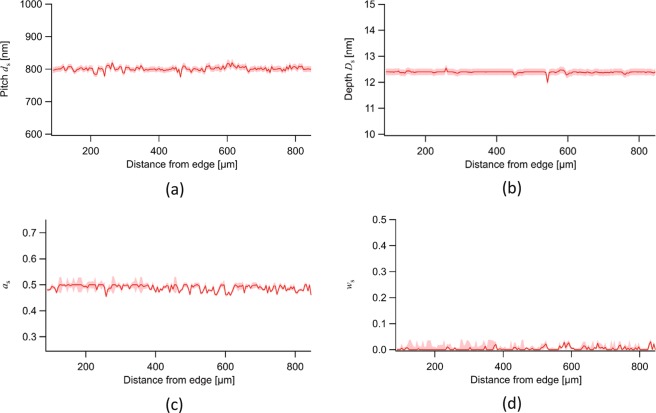
Real-space distributions of (**a**) *d*_s_, (**b**) *D*_s_, (**c**) *a*_s_, and (**d**) *w*_s_ determined by least-squares fitting to glancing-angle dependence of $\tilde{{\mathscr{V}}}(x,{y}_{{\rm{c}}})$ for ${p}_{{\rm{s}}}{d}_{1}=400\,{\rm{nm}}$ and *p*_s_*d*_1_ dependence of $\tilde{{\mathscr{V}}}(x,{y}_{{\rm{c}}})$ for glancing angle of 0.180°.

In our approach, the real-space distribution of structural parameters can be obtained. Figure 6(a–d) show the real-space distributions of *d*_s_, *D*_s_, *a*_s_, and *w*_s_ determined by least-squares fitting. Here, *a*_s_ was determined under a constraint of ${a}_{{\rm{s}}}\le 0.5$ because both the dependences are symmetric around ${a}_{{\rm{s}}}=0.5$ and only the deviation of *a*_s_ from 0.5 can be determined from the least-squares fitting. The reduced chi square for the least-squares fittings in this region was 1.20 ± 0.29.

The parameters *d*_s_, *D*_s_, and *a*_s_ for the best-fit curves were almost the same as those determined using an atomic force microscope (AFM), a transmission electron microscope (TEM) (Supplementary Note Section 2), and a Bonse-Hart camera^19^ enabling us to measure angular distribution of grazing-incidence ultra-small-angle X-ray scattering (GIUSAXS) intensity, but *w*_s_ for the best-fit curve was substantially different from the results of the AFM and GIUSAXS measurements. The discrepancy with the result of AFM should be attributed to the effect of the shape of the AFM tip used. The discrepancy with the result of the GIUSAXS measurement should be because GIUSAXS dispersed in the surface normal direction cannot be distinguished with a Bonse-Hart camera. In particular, the width of the rocking curve obtained with a Bonse-Hart camera is broadened by the dispersion of the GIUSAXS in the surface normal direction, and this broadening makes interpretation of the curve difficult. The proposed method detects GISAXS with the same momentum transfer in the surface normal direction as that of the specularly reflected X-rays and finite in-plane scattering vector components, and distinguish it from both the specularly reflected X-rays and the other GISAXS. This facilitates the interpretation of experimental data. For example, from the width of the rocking curve of ${\mathscr{V}}(x,{y}_{{\rm{c}}})$ when the sample is rotated around the surface normal, the undulation of the lines was estimated to be less than 0.1°, while the width of rocking curve obtained with a Bonse-Hart camera was 1.63°^19^, which was likely due to the effect of the grating truncation rods intersecting with the Ewald sphere^27^.

It is expected that one-dimensional periodic surface and interface structures with some small randomness, such as the undulation of the lines, generally increase the minimum values of the normalized visibility in the glancing-angle and ${p}_{{\rm{s}}}{d}_{1}$ dependences because the scattering power of the GISAXS from the surface and interface structures becomes smaller due to the randomness. Because the randomness changes only the minimum values and does not affect the shapes of the glancing-angle and ${p}_{{\rm{s}}}{d}_{1}$ dependences, we can determine average values of *D*_s_, *a*_s_, *w*_s_, *σ*_D_, and *d*_s_ at each pixel. Two-dimensional periodic surface and interface structures should also change only the minimum values because of the reduced scattering power, but we can distinguish this change from that due to the randomness by rotating two-dimensional structures 90° around the surface normal and measuring the glancing-angle and ${p}_{{\rm{s}}}{d}_{1}$ dependences.

In conclusion, we developed an approach based on X-ray grating interferometry for investigating real-space variations in surface and interface morphology through wavefront analysis. We successfully demonstrated surface structure analysis for a SiO_2_ line and space pattern with a depth of 10 nm.

Theoretical descriptions of grating-based X-ray imaging^12,16^ support that our approach can be used for distinguishing GIUSAXS from specular reflection in grazing incidence geometry. Because GIUSAXS has a very small scattering angle (typically of the order of 10 *μ*rad), it is generally not easy to distinguish between GIUSAXS and specularly reflected X-rays. Our approach can enable GIUSAXS to be distinguished even without using a parallel X-ray beam — it works even with an X-ray beam with a wide angular spread from a low-brilliance laboratory X-ray source if the Talbot-Lau type X-ray grating interferometry is employed^21^.

Our approach can be applied to surface and interface structure analysis as long as the scattering power of the GISAXS from the structure in the lateral direction is sufficiently strong to reduce the visibility of the self-image. Because X-ray grating interferometry allows for large-field-of-view (even 100 mm-width) imaging using commercially available gratings, a large-size sample can also be measured by our approach although a larger sample in the direction of the optical axis makes the resolution of ${p}_{{\rm{s}}}{d}_{1}$ lower. This merit should make it possible to realize *in situ* observation of surface and interface phenomena such as capillarity, wetting, and tribological phenomena, which generally require various sample environments.

Innis-Samson *et al*. recently achieved tomographic reconstruction of two-dimensional X-ray reflectivity by rotating a sample around the surface normal. However, GIUSAXS could not be distinguished from the specularly reflected X-rays they measured^28^. Scanning GISAXS tomography was also achieved for isotropic surface structures in the lateral direction^29–32^. Our approach using an X-ray sheet beam is suitable for structural parameter mapping with a large field-of-view, which is not covered with coherent X-ray diffraction imaging^33,34^. It will enable tomographic reconstruction of two-dimensional structural parameters from not only GIUSAXS-eliminated X-ray reflectivity but also GISAXS. Because a white synchrotron X-ray beam enables ms-order X-ray tomography with X-ray grating interferometry^35–37^, our approach should be potentially applicable to dynamic research on surface and interface morphology. Note that grazing-incidence small-angle neutron (GISANS) imaging and tomography is also enabled by the use of neutron grating interferometry^38–41^. Thus, our approach has the potential to become a powerful tool for investigating real-space variations in surface and interface morphology.

## Methods

The experiment was performed at the BL20XU beamline in SPring-8, Japan, where a high-spatial-coherence synchrotron X-ray beam monochromatized by a cooled Si 111 double crystal monochromator is available at an experimental station located 245 m downstream of an undulator source^42^. The energy of the X-ray beam was fixed at 9.000 ± 0.001 keV. The size of the front-end slit in front of the monochromator was set to be 0.2 mm (horizontal) × 0.1 mm (vertical). We used a *π*/2-phase (5.9 *μ*m-depth) Si grating with an average pitch of 34.7 *μ*m, which was fabricated by ultraviolet lithography and deep reactive ion etching. The thickness of the substrate of the phase grating was reduced to 50 *μ*m so as to increase the intensity of the transmitted X-rays. The phase grating was located at a distance of 214 m downstream of the front-end slit. The lines of the grating were aligned in the vertical direction.

An X-ray camera consisting of a phosphor screen (10 *μ*m-P43, Gd_2_O_2_S: Tb+ fine powders), a relay lens, and a scientific complementary metal-oxide-semiconductor (sCMOS) camera (Hamamatsu Photonics ORCA Flash 4.0) was used as the detector. The detector was located 4.458 m from the grating, where the Talbot order for the self-image is 0.5 and its visibility is maximized. The effective pixel size of the detector was 4.4 *μ*m, and the average full width at half maximum (FWHM) of the point spread function of the detector was 14.6 ± 0.6 *μ*m, which is sufficiently high to resolve a self-image of the phase grating. The small pincushion distortion of the detector was corrected, enabling the surface curvature of the sample to be obtained precisely.

We used the sample with a 400 nm line and space reported in a previous paper^19^, where conventional X-ray reflectivity measurement and GIUSAXS measurement with a Bonse-Hart camera were used for characterizing it. A one-side polished 525 *μ*m-thick Si(001) wafer was used for the sample. The wafer was thermally oxidized to form a 200 nm-thick SiO_2_ layer on its surface, and cut into 12 mm × 12 mm chips. The 400 nm line and space pattern on the sample was fabricated by electron beam lithography and inductively coupled plasma (ICP) etching with C_3_F_8_, O_2_, and Ar gases (etching rate: 0.1 nm/s) in an area of 5 mm × 5 mm on one of the chips.

The sample was positioned between the grating and the detector and its surface was illuminated by a 4 mm (horizontal) × 0.28 mm (vertical) X-ray sheet beam. By changing the position of the sample, we were able to change *p*_s_ defined in Eq. (5). The sample position was determined by the glancing-angle dependence of the position of the specularly reflected X-ray beam. The error bars for the pixels in the obtained images were determined using a previously proposed method^43^, and the low signal-to-noise ratio data following a Rician distribution^44^ were not used for the least-squares fittings. Note that we can extend the range of the glancing angle shown in Fig. 3(a–c) simply by making the measurement time longer and/or using a higher signal-to-noise-ratio photon-counting X-ray detector.

In the simulation and least-squares fittings, the projection approximation was used, *i*.*e*. the effect of Fresnel diffraction by the sample was neglected. Using projection approximation enabled the calculation time to be reduced with negligible loss of precision^16^.

## Supplementary information

Supplementary Movie 1

Supplementary Movie 2

SUPPLEMENTARY INFORMATION

## Data Availability

The datasets generated during and/or analysed during the current study are available from the corresponding author on reasonable request.
