# Supplementary material for: Probing Surface Morphology using X-ray Grating Interferometry
Source: Sci Rep. 2019 Oct 1;9:14120. doi: 10.1038/s41598-019-50486-5 (PMC6773752; doi:10.1038/s41598-019-50486-5)
Supplement: Supplementary file 3 — SUPPLEMENTARY INFORMATION [file 41598_2019_50486_MOESM3_ESM.pdf]

SUPPLEMENTARY INFORMATION

Probing Surface Morphology using X-ray Grating Interferometry

Wataru Yashiro[1,*], Susumu Ikeda[2], Yasuo Wada[3], Kentaro Totsu[4], Yoshio Suzuki[5], and Akihisa Takeuchi[5]

[1]Institute of Multidisciplinary Research for Advanced Materials (IMRAM), Tohoku University, 2-1-1 Katahira, Aoba-ku, Sendai, Miyagi 980-8577, Japan

[2]WPI-Advanced Institute for Materials Research (WPI-AIMR), Tohoku University, 2-1-1 Katahira, Aoba-ku, Sendai, Miyagi 980-8577, Japan

[3]Faculty of Science and Technology, Keio University, 3-14-1 Hiyoshi, Kohoku-ku, Yokohama, Kanagawa 223-8522, Japan

[4]Micro System Integration Center (mSIC), Tohoku University, 519-1176 Aramaki-Aza-Aoba, Aoba-ku, Sendai, Miyagi 980-0845, Japan

[5]Japan Synchrotron Radiation Research Institute (JASRI), 1-1-1 Kouto, Sayo-cho, Sayo-gun, Hyogo 679-5198, Japan

*wyashiro@tohoku.ac.jp

SUPPLEMENTARY NOTE

1. Results of numerical calculations for normalised visibility $\mathcal{V}$

Here, we show the dependences of normalised visibility $\mathcal{V}$ on the structural parameters of the sample shown in Fig. 5 (a) and the width of the point spread function (PSF) of the image detector.

1.1 Glancing-angle dependence of $\mathcal{V}$

Figures S1, S2, S3, S4, and S5 plot the glancing-angle dependences of $\mathcal{V}$ for $p_s d_1$ = 400 nm.

Figure S1 shows the glancing-angle dependence of $\mathcal{V}$ on D_s for d_s = 800 nm, a_s = 0.5, w_s = 0, and σ_D = 6 μm, where $\sigma_D \equiv W_D/(2\sqrt{2\ln 2})$. The glancing-angle dependence of $\mathcal{V}$ sensitively changes the fringe period of $\mathcal{V}$. These fringes are similar to the Kiessig fringes in X-ray reflectivity curves but have a different origin: $\mathcal{V}$ is determined by the autocorrelation in the lateral direction.

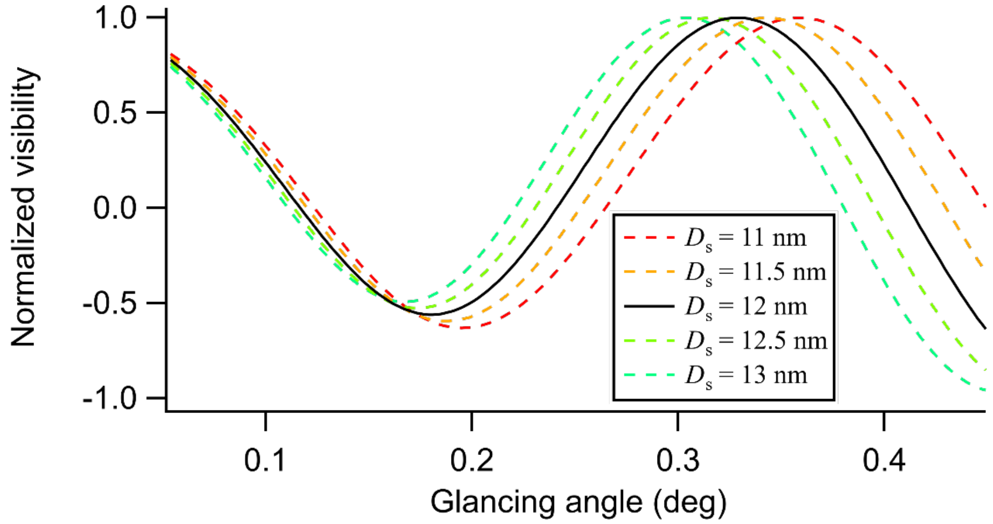

Figure S1. Glancing-angle dependence of $\mathcal{V}$ on D_s for d_s = 800 nm, a_s = 0.5, w_s = 0, and σ_D = 6 μm, where $\sigma_D \equiv W_D/(2\sqrt{2\ln 2})$.

Figure S2 shows the glancing-angle dependence of $\mathcal{V}$ on a_s for d_s = 800 nm, D_s = 12 nm, w_s = 0, and σ_D = 6 μm, and Fig. S3 shows it on w_s for d_s = 800 nm, D_s = 12 nm, a_s = 0.5, and σ_D = 6 μm. A deviation of a_s from 0.5 reduced the amplitude of the fringes of normalised visibility while a change in w_s caused a shift in the dip position of the normalised-visibility fringes.

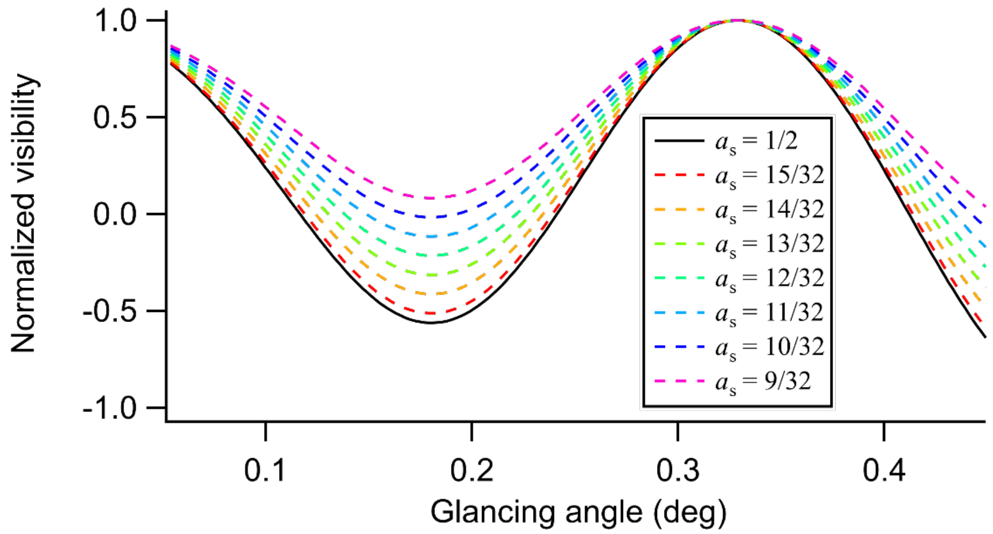

Figure S2. Glancing-angle dependence of $\mathcal{V}$ on a_s for d_s = 800 nm, D_s = 12 nm, w_s = 0, and σ_D = 6 μm.

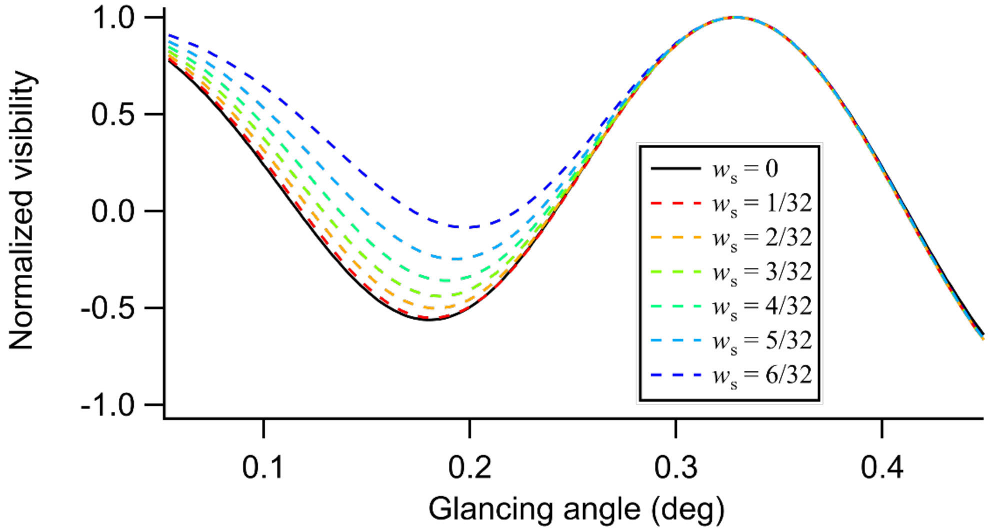

Figure S3. Glancing-angle dependence of $\mathcal{V}$ on w_s for d_s = 800 nm, D_s = 12 nm, a_s = 0.5, and σ_D = 6 μm.

Figure S4 shows the glancing-angle dependence of $\mathcal{V}$ on σ_D for d_s =800 nm, D_s = 12 nm, a_s = 0.5, and w_s = 0. The minimum value of the normalised visibility changed while the effect of the blur due to a finite value of σ_D decreased as the glancing angle increased.

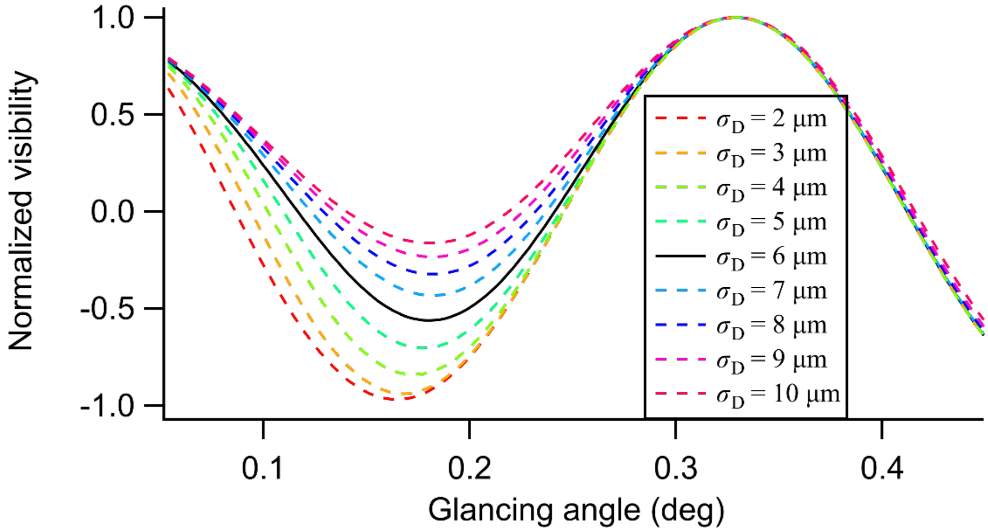

Figure S4. Glancing-angle dependence of $\mathcal{V}$ on σ_D for d_s = 800 nm, D_s = 12 nm, a_s = 0.5, and w_s = 0.

Figure S5 (a) and (b) shows the glancing-angle dependence of $\mathcal{V}$ on d_s for D_s = 12 nm, a_s = 0.5, w_s = 0, and σ_D = 6 μm; $\mathcal{V}$ was not sensitive to d_s. As shown below, the $p_s d_1$ dependence on d_s at a fixed glancing angle is much more sensitive than the glancing angle dependence.

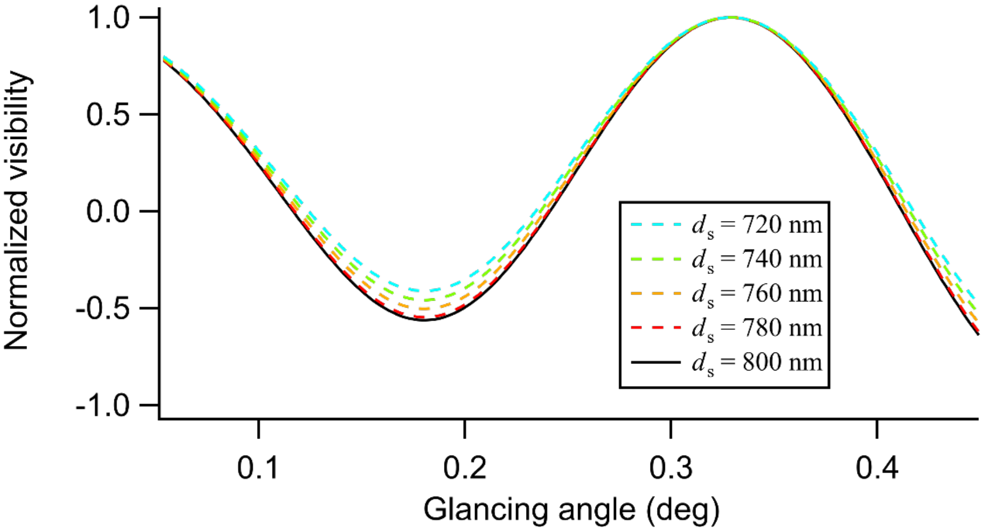

(a)

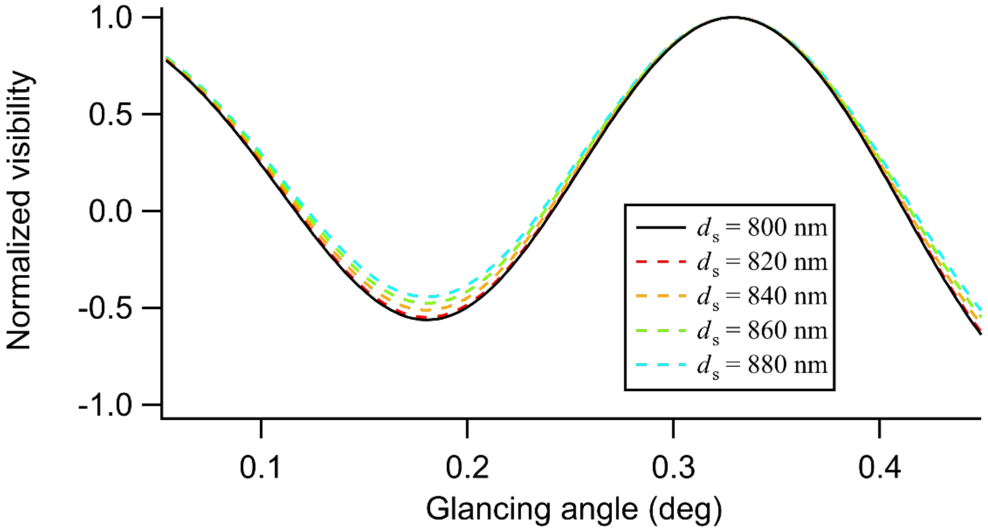

(b)

Figure S5. Glancing-angle dependence $\mathcal{V}$ on d_s for D_s = 12 nm, a_s = 0.5, w_s = 0, and σ_D = 6 μm ((a) d_s = 720, 740, 760, 780, and 800 nm. (b) d_s = 800, 820, 840, 860, and 880 nm).

1.2 $p_s d_1$ dependences of $\mathcal{V}$

Figures S6, S7, S8, S9, and S10 plot the $p_s d_1$ dependences of $\mathcal{V}$ for a glancing angle of 0.180°.

Figure S6 shows the $p_s d_1$ dependence of $\mathcal{V}$ on d_s for D_s = 12 nm, a_s = 0.5, w_s = 0, and σ_D = 6 μm, where $\sigma_D \equiv W_D/(2\sqrt{2\ln 2})$. The position of the peak and dip sensitively shifted.

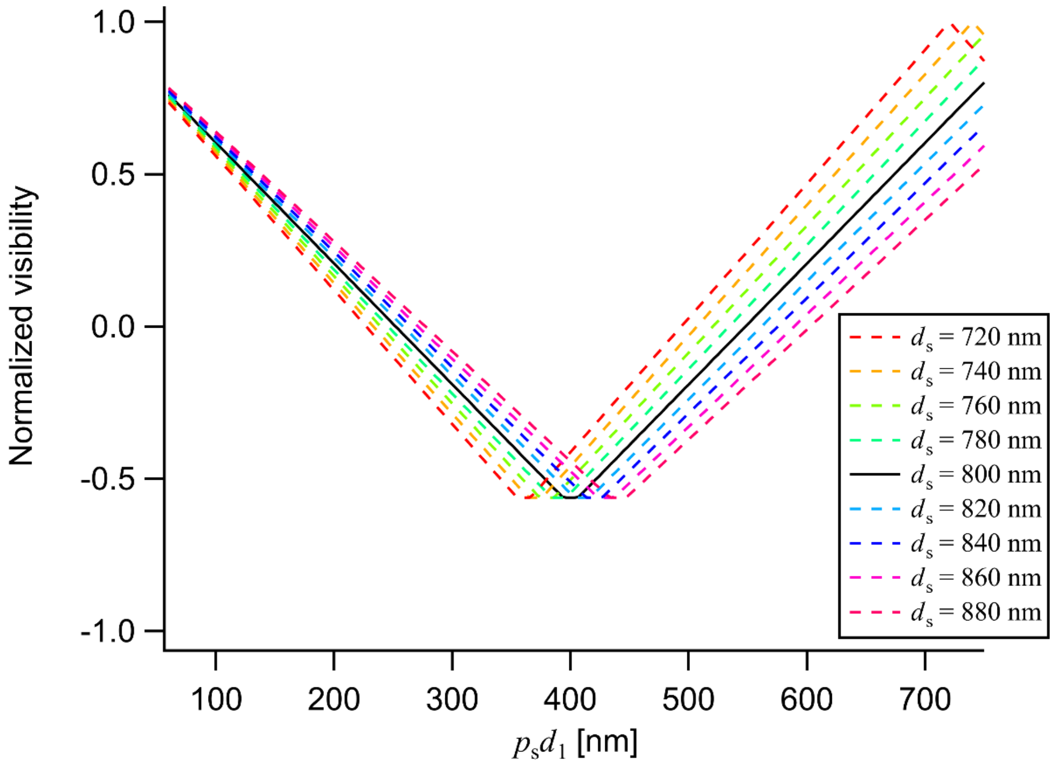

Figure S6. $p_s d_1$ dependence of $\mathcal{V}$ on d_s for D_s = 12 nm, a_s = 0.5, w_s = 0, and σ_D = 6 μm.

Figure S7 shows the $p_s d_1$ dependence of $\mathcal{V}$ on a_s for d_s = 800 nm, D_s = 12 nm, w_s = 0, and σ_D = 6 μm, and Fig. S8 shows it on w_s for d_s = 800 nm, D_s = 12 nm, a_s = 0.5, and σ_D = 6 μm.

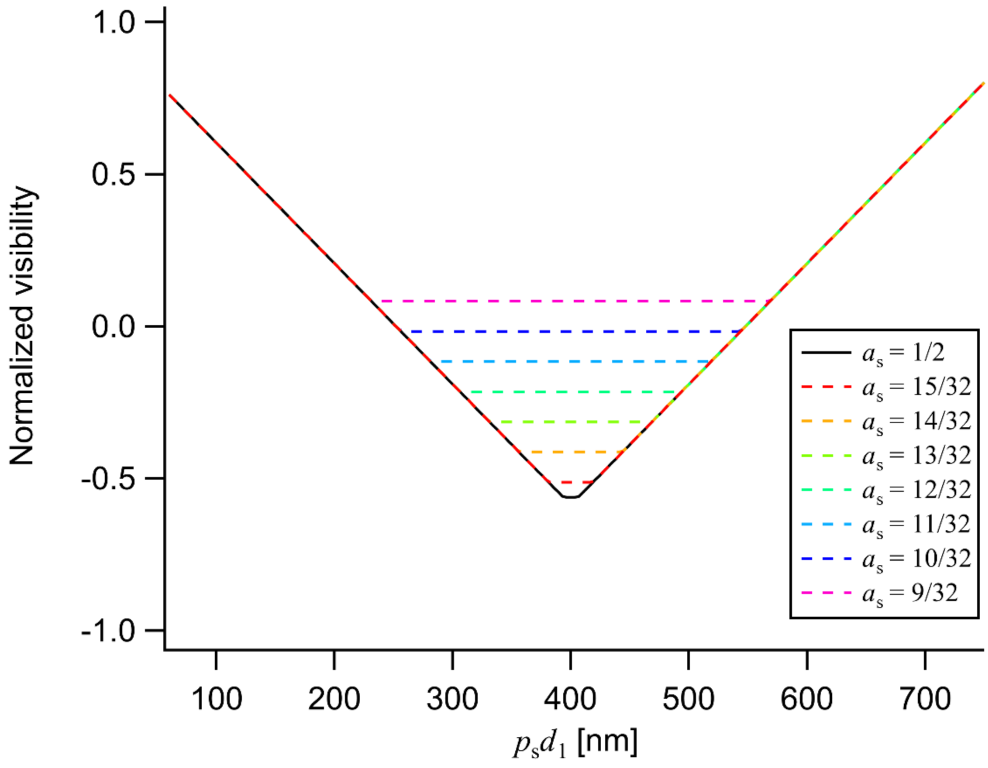

Figure S7. $p_s d_1$ dependence of $\mathcal{V}$ on a_s for d_s = 800 nm, D_s = 12 nm, w_s = 0, and σ_D = 6 μm.

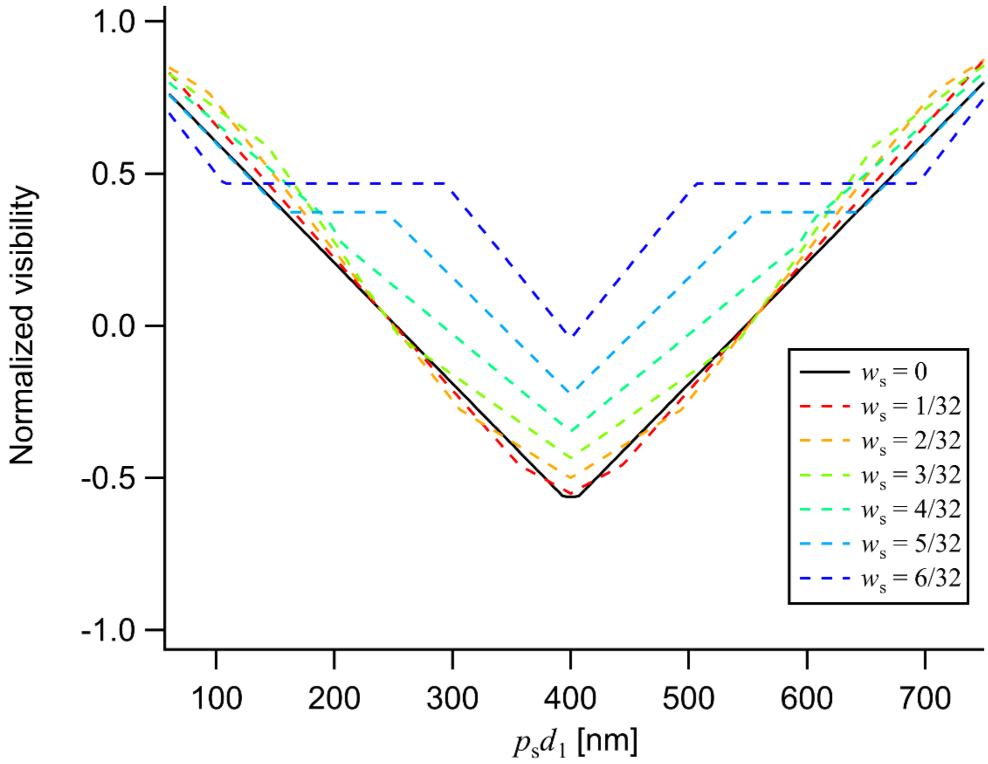

Figure S8. $p_s d_1$ dependence of $\mathcal{V}$ on w_s for d_s = 800 nm, D_s = 12 nm, a_s = 0.5, and σ_D = 6 μm.

Figure S9 shows the $p_s d_1$ dependence of $\mathcal{V}$ on σ_D for d_s = 800 nm, D_s = 12 nm, a_s = 0.5, and w_s = 0; the minimum position was not changed but the minimum value of the normalized visibility was sensitive to σ_D.

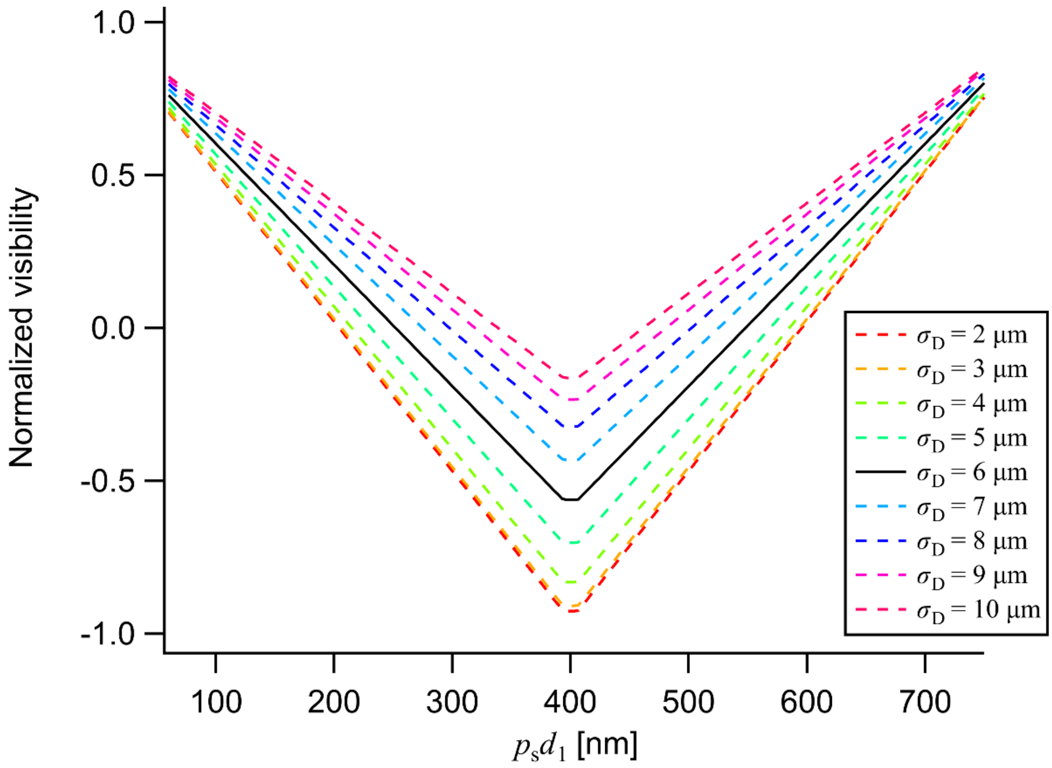

Figure S9. $p_s d_1$ dependence on σ_D for d_s = 800 nm, D_s = 12 nm, a_s = 0.5, and w_s = 0.

Figure S10 shows the $p_s d_1$ dependence of $\mathcal{V}$ on D_s for d_s = 800 nm, a_s = 0.5, w_s = 0, and σ_D = 6 μm; $\mathcal{V}$ was not sensitive to D_s.

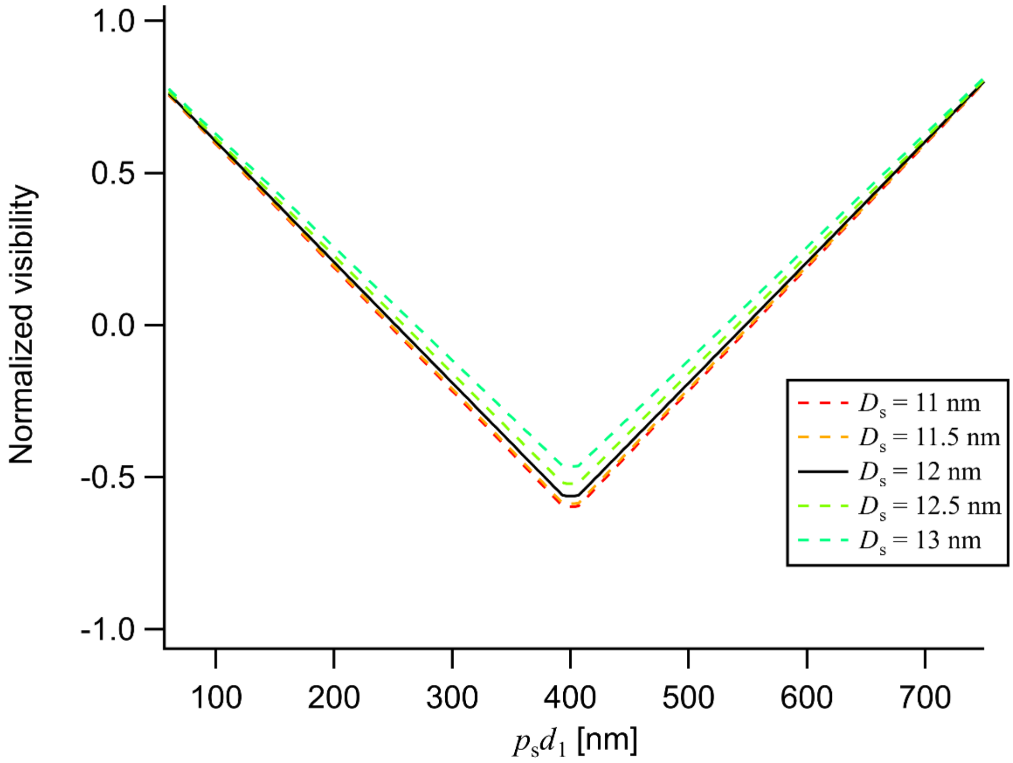

Figure S10. $p_s d_1$ dependence $\mathcal{V}$ on D_s for d_s = 800 nm, a_s = 0.5, w_s = 0, and σ_D = 6 μm.

2. Results of other experimental techniques

Here, we show the results of atomic force microscopy (AFM), transmission electron microscopy (TEM), and three-dimensional optical profilometry for the sample obtained after the experiment of X-ray grating interferometry.

2.1 AFM

Figure S11 shows an image of the sample surface obtained by AFM (nanocute, Hitachi High-Technologies Corporation) in the tapping-mode operation with a resonant frequency of 400 kHz. A cantilever with a spring constant of 40 N/m was used.

Although AFM can provide only local structural information, the structural parameters d_s, D_s, and a_s (defined in Fig. 5 (a)) obtained from the AFM image were consistent with those obtained from our technique and TEM shown below. The slop width $2d_s w_s$ was on the order of a few tens of nm, which was one order of magnitude larger than those obtained from our technique and TEM. The discrepancy of w_s should be attributed to the effect of the shape of the AFM tip used.

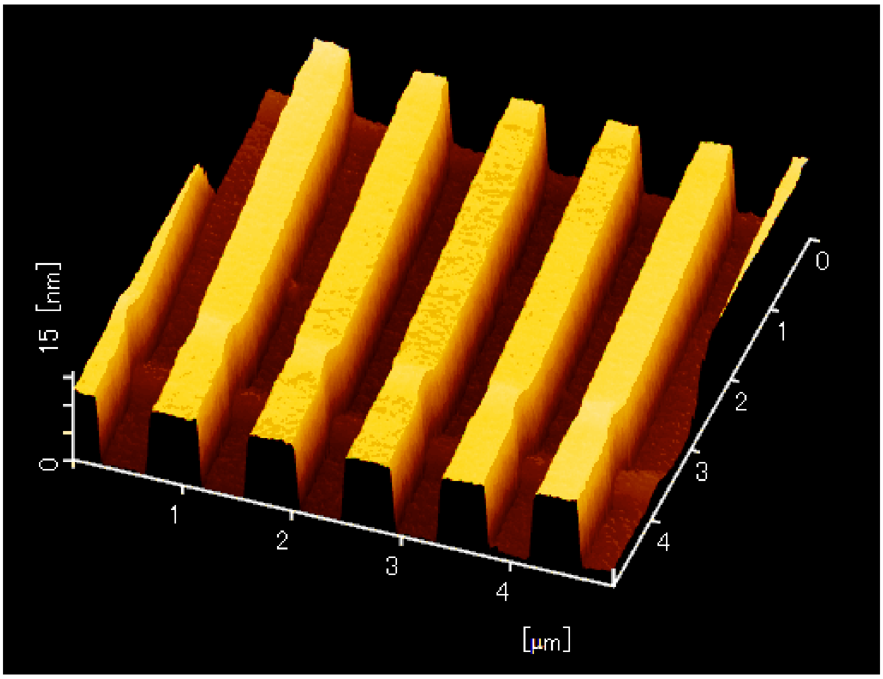

Figure S11. Image of sample surface obtained by atomic force microscopy.

2.2 TEM

Figure S12 shows a sectional image of the sample obtained by TEM (JEM-2100F, JEOL Ltd.) operated with an acceleration voltage of 200 kV.

Although TEM requires sample destruction and provides only local structural information, the structural parameters d_s, D_s, a_s, and w_s (defined in Fig. 5 (a)) obtained from the TEM image were consistent with those obtained from our technique.

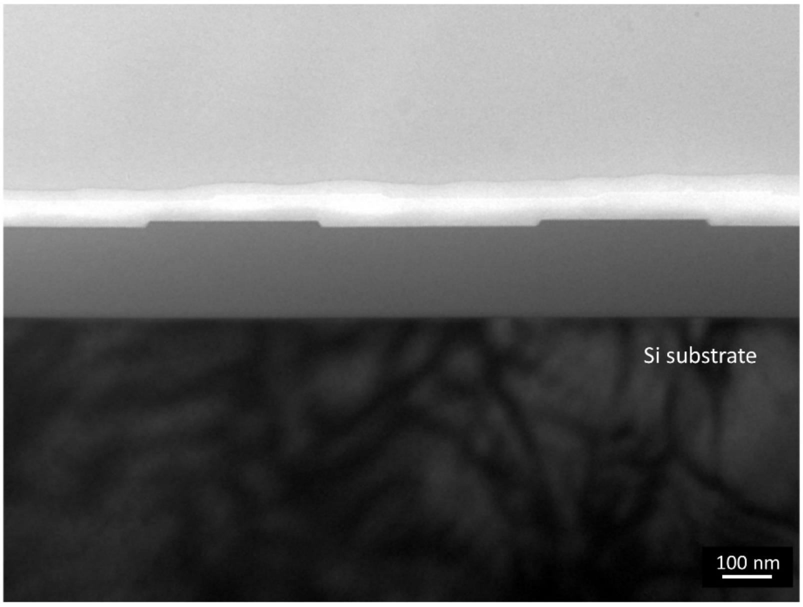

Figure S12. Sectional image of the sample obtained by transmission electron microscopy.

2.3 Three-dimensional optical profilometry

The upper figure of Fig. S13 shows the surface image of the sample obtained by three-dimensional optical profilometry (NexView^{TM}, ZYGO), and the lower figure shows a line profile along a diagonal line (dashed line in the upper figure).

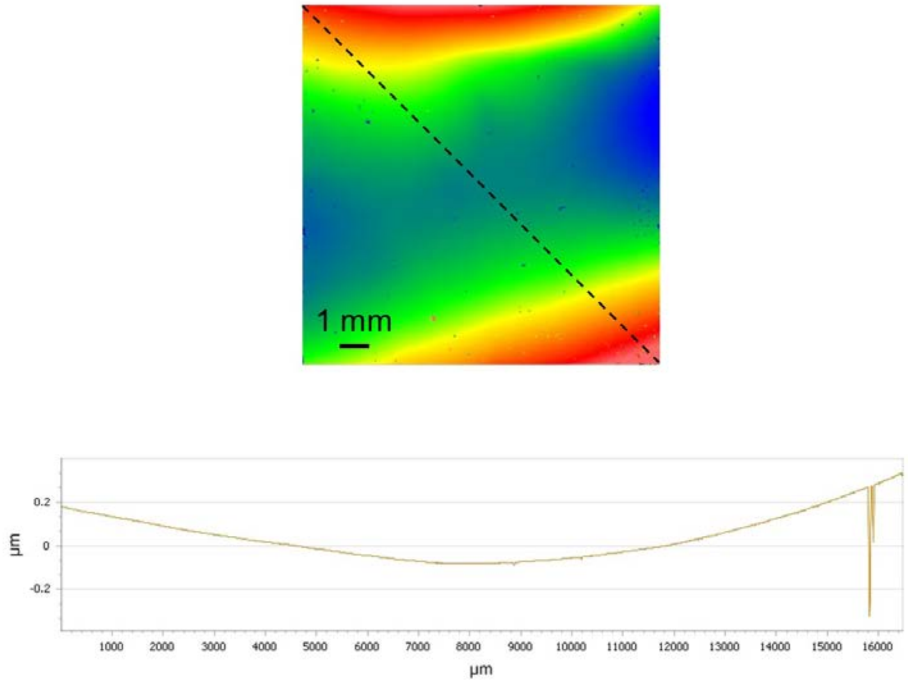

Figure S13. Surface image of the sample obtained by three-dimensional optical profilometry (upper) and line profile along diagonal line (lower).

3. X-ray reflectivity

Figure S14 shows $\mathcal{I}$ obtained from Fig. 3 (a). The red crosses in the figure are $\mathcal{I}$ in the region with the line and space pattern averaged over 100 pixel in the lateral direction, while blue filled circles are those in the region without the line and space pattern. The black solid curve was calculated from the Fresnel equation for a flat surface of SiO_2 (2.3 g/cm^3). Note that the behavior of $\mathcal{I}$ below the critical angle was explained with an effective width of the PSF for the 100-pixel area and the Fresnel diffraction by the sample taken into account.

It can be seen that the difference between the two experimental curves is negligible and they agree well with the calculated curve for a flat SiO_2 surface. In addition, no clear Kiessig fringes can be seen in the X-ray reflectivity curve for the region with the line and space pattern.

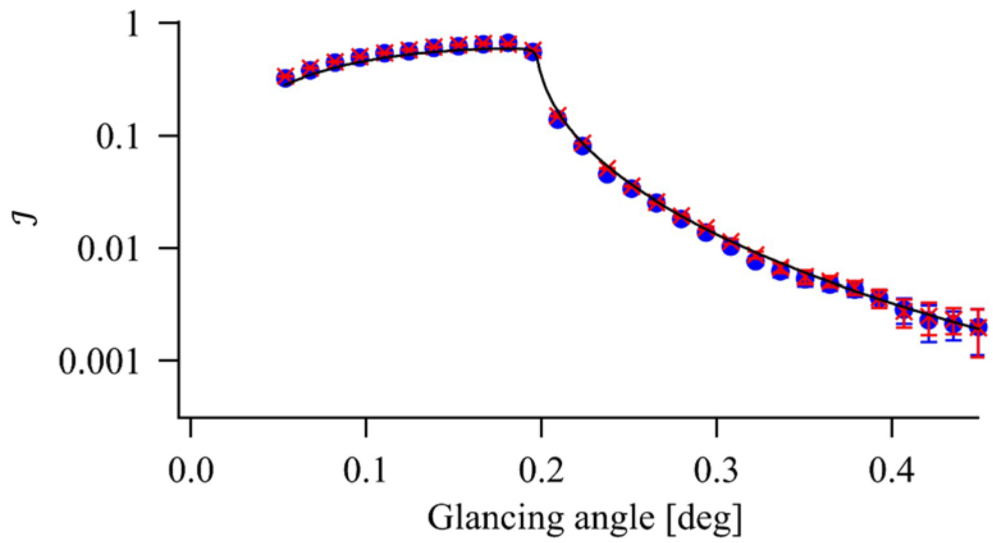

Figure S14. $\mathcal{I}$ obtained from Fig. 3 (a) (red crosses: $\mathcal{I}$ in the region with the line and space pattern averaged over 100 pixel in the lateral direction, blue filled circles: $\mathcal{I}$ in the region without the line and space pattern, black solid curve: calculated X-ray reflectivity from the Fresnel equation for a flat SiO_2 surface.
